# Evolution of nutritional status of infants infected with the human immunodeficiency virus

**DOI:** 10.1590/S1516-31802000000500007

**Published:** 2000-09-01

**Authors:** Vânia Aparecida Leandro-Merhi, Maria Marluce dos Santos Vilela, Marcos Nolasco da Silva, Fábio Ancona Lopez, Antônio de Azevedo Barros

**Keywords:** Growth and development, Nutritional status, Children, HIV, Acquired immunodeficiency syndrome, Crescimento e desenvolvimento, Estado nutricional, Crianças, HIV, Síndroma da imunodeficiência adquirida

## Abstract

**CONTEXT:**

There are today only a limited number of studies defining growth parameters and nutritional status for HIV children.

**OBJECTIVE:**

To study the nutritional status of infants infected with the human immunodeficiency virus.

**TYPE OF STUDY:**

Longitudinal study.

**SETTING:**

Department of Pediatrics, Faculty of Medical Sciences, UNICAMP, Campinas, Brazil.

**PARTICIPANTS:**

One hundred and twenty-four children born to HIV infected mothers were evaluated from birth until the age of two years. They were subdivided into two groups: 71 infected children and 53 non-infected children.

**MAIN MEASUREMENTS:**

Growth was evaluated in both groups by comparing Z-scores for weight/age (W/A), length/age (H/A) and weight/length (W/H) (using the NCHS curves as reference).

**RESULTS:**

The Z-score analyses showed that there was a significant difference between the two groups for all the variables studied, except for the H/A value at 3 months of age and the W/H value at 21 months of age, which showed P > 0.05.

**CONCLUSIONS:**

The growth of infected infants was observed to be severely affected in comparison with that of seroreversed infants in the same age groups. Although clinical manifestations may take time to appear, the onset of growth changes begin soon after birth.

## RESUMO

**CONTEXTO:**

Atualmente há estudos limitados que definem parâmetros de crescimento e estado nutricional em crianças com HIV.

**OBJETIVO:**

Estudar o estado nutricional de crianças infectadas com o vírus da imunodeficiência humana.

**TIPO DE ESTUDO:**

Estudo longitudinal.

**LOCAL:**

Serviço de imunodeficiência pediátrica da Universidade Estadual de Campinas, Campinas, Brasil.

**PARTICIPANTES:**

124 crianças nascidas de mulheres infectadas com o HIV foram avaliadas desde o nascimento até dois anos de idade. Elas foram subdivididas em dois grupos: 71 crianças infectadas e 53 crianças não-infectadas.

**PROCEDIMENTOS:**

A avaliação do estado nutricional foi realizada pela comparação do Z-score para peso/idade(P/I),altura/idade(A/I) e peso/altura(P/A),usando as curvas do NCHS como referência. Para a comparação do Z-score foram utilizados os testes T de Student e U de Mann-Whitney. Os testes foram conduzidos ao nível de significância=5%.

**RESULTADOS:**

A análise do Z-score revelou que houve evidência de diferença entre os dois grupos, para todas as variáveis estudadas, exceto o índice de A/I aos 3 meses e P/A aos 21 meses.

**CONCLUSÕES:**

O estado nutricional das crianças infectadas foi grave-mente afetado em comparação com as crianças sororreversoras no mesmo grupo de idade. Embora as manifestações clínicas possam demorar a aparecer, as alterações no crescimento surgem logo após o nascimento.

## INTRODUCTION

AIDS has caused a tremendous impact ever since the first adult and child cases were reported at the Center for Disease Control, Atlanta, USA^1^ and has become one of the greatest health problem all over the world. According to the estimates in 1994, there were approximately 2 million children infected with HIV in the world. In the United States of America, there are 12,000 infected children.^2^

As the number of heterosexual men infected increased, the number of women and children infected also increased. In 1985, the highest number of AIDS cases reported was among homosexuals followed by bisexuals, drug addicts and lastly heterosexuals. In 1987, most of the cases reported were heterosexuals, followed by homosexuals and endovenous drug addicts.^3^ Consequently, the number of cases of AIDS in children increased. There was an improvement in the notification of cases when pre-natal follow-up of the mothers was conducted with later follow-up of the children. In comparison with the 1990's, the number of infected women has increased and as a result the number of infected children has also risen. In September 1993, there were 4,906 pediatric cases of AIDS diagnosed in the United States – 88% were due to perinatal transmission from the infected mother and 11% were as a result of contaminated blood transfusions.^4^ According to the World Health Organization, by the year 2000, approximately 40 million adults and 2 million children will have been infected with HIV.^5^

In Brazil, the first cases of AIDS in children were reported in 1983 and almost all of them were infected by blood transfusions or by the transfusion of its derivatives.^3^

Up to August 1998, there had been 140,362 cases of AIDS reported in Brazil. Of these, 4815 (3.4%) were children below the age of 13, which demonstrated that in this age group there had been an increase in this disease. Among infant cases, 3825 (79.4%) acquired the disease through vertical transmission, 205 (4.3%) were hemophiliacs, 279 (5.8%) acquired it through transfusions and 489 (10.2 %) from unknown sources, and 1986 (41.2%) of these infant cases had died.^3^

The clinical manifestations generally shown by such children are: generalized lymphadenopathy, hepatosplenomegaly, oral candidiasis and recurring infections.^6–9^

There are today only a limited number of studies defining growth parameters for HIV children.^10,11^ Halsey, et al.^12^ (1990), studied a population sample in Haiti and found that the weight of the babies born to mothers positive for HIV type were similar, independent of the state of infection during that period, but different from the weight of babies born to mothers negative for HIV type. Although the ingestion of nutrients and the body mass were not reported, the weight of children positive for HIV at 3 months differed from the weight of children seronegative for HIV and the HIV negative control group. Miller, et al.^10^ (1993) observed that at birth, normal children and HIV infected children had similar weight, gestational age and percentile weight but at between 19 and 21 months, their percentile weights differed significantly and the lengths remained the same. McKinney, et al.^11^ (1993) analyzed the growth rate of children during the first two years of life and found that the growth index of HIV infected children during the first four months was significantly less than that of non-infected children regarding weight as well as length in relation to age and that the linear growth as well as the gain in weight were proportionally reduced. Moye, et al.^13^ (1996) studied the magnitude of the infection caused by the human immunodeficiency virus, acquired either perinatally or congenitally, on somatic growth from birth up to the age of 18 months. They concluded that the infected children showed a progressive decline in body mass index from birth to the age of 6 months.

Growth monitoring is an important tool for evaluating the child's health. We were able to study this question profoundly because of the information available regarding the weight and length of seroreversed infected children, who were being treated at the Outpatient Pediatrics Department, Hospital das Clínicas, State University of Campinas, SP, Brazil. The objective of this research was to study the nutritional status with regard to weight and length of children with the human immunodeficiency virus aged zero to 24 months, and to compare these results with those of non-infected children with mothers who were positive for HIV.

## METHODS

The procedures that follow were in accordance with the ethical standards of the committee responsible for human experimentation and with the Helsinki declaration of 1975, as revised in 1993.

### Design

A mixed longitudinal study was conducted during the follow-up period from August 1985 to April 1996.

### Setting

Department of Pediatrics, State University of Campinas.

### Participants

Children whose mothers were found to be positive for HIV at the Outpatient Department, Pediatric Immunodeficiency Service, Hospital das Clínicas, State University of Campinas. The following variables were analyzed: age, sex, gestational age at birth, weight at birth, weight and length during the follow-up period. The study was conducted on 124 patients who fulfilled the criteria established. Children who weighed less than 2500g and those who started treatment after the age of two years were excluded.

The criteria for inclusion were:

- Born after full gestational term and with weight ≥ 2500 g.^16^
- Absence of congenital diseases that could interfere in the development of the child and its nutritional state.
- Only vertically infected children were included (mother → child).

The children were classified into two groups:

- *Infected Children:* those children who were serologically positive for the human immunodeficiency virus after conducting the enzyme-linked immunoabsorbent assay (ELISA) and confirmed by the Western Blot or Immunofluorescence test after the age of 18 months or before this date, if the child showed symptoms of the disease.
- *Non-infected Children (seroreversed):* the children were considered to be non-infected when they showed a complete reversal of the serological results (ELISA) up to 18 months of age, with normal immunology and without any symptoms of the human immunodeficiency virus infection.

### Main Measurements

*Anthropometry.* A nutritional evaluation was conducted with the help of weight and length measurements and utilizing the curves of the “National Center for Health Statistics”,^14^ which have been recommended by the World Health Organization (WHO). The relationships weight/length, length/age and weight/age were expressed in terms of “Z-scores”.^15^ In order to analyze the Z scores, children between the ages of zero and 24 months and infected with the human immunodeficiency virus were chosen, and also children between zero and 24 months who were seropositive at the beginning and became negative by the age of 18 months.

### Statistical Methods

The Student T test was used in the case of independent samples when the normality supposition was satisfied and the Mann-Whitney U test was used for the rest. The tests were conducted for a 5% significance (α) level.

The patients were classified according to their ages for statistical analysis:

- Between 0 and 6 months: The measurements closest to three months were taken for the calculation of the Z-scores.
- Between 6 and 12 months: The measurements closest to nine months were taken for the calculation of the Z-scores.
- Between 12 and 18 months: The measurements closest to fifteen months were taken for the calculation of the Z-score.
- Between 18 and 24 months: The measurements closest to twenty-four months were taken for the calculation of the Z-score.

Z-scores were calculated for weight/age, length/age and weight/length in the cases of both the infected and the non-infected groups of children.

## RESULTS

The children were divided into two groups – infected and non-infected – and classified according to their sex, color, mother's education, type of birth and whether they were breast-fed (Table 1).

**Table 1:** Characteristics of the population studied

Characteristics	Children					
	Infected (n = 71)		Non-infected (n = 53)		Total (n = 124	
	No.	%	No.	%	No.	%
Sex						
Male	34	47.9	31	58.5	65	52.4
Female	37	52.1	22	41.5	59	47.6
Situation						
Follow-up	32	45.1	30	56.6	62	50
Discharged	-	-	12	22.6	12	9.7
Died(*)	23	32.4	-	-	23	18.5
Abandoned	13	18.4	10	18.8	23	18.6
Transferred	3	4.2	1	1.9	23	3.2
Mother's education (**)						
Middle school complete	5	9.4	-	-	5	5.7
Middle school incomplete	35	66.0	27	79.4	62	71.3
High school complete	6	11.3	4	11.8	10	11.5
High School incomplete	3	5.7	2	5.9	5	5.7
University	4	7.5	1	2.9	5	5.7
Childbirth (***)						
Cesarean	23	35.9	19	38.8	42	37.2
Vaginal	41	64.1	30	61.2	71	62.8
Breast feeding (****)						
Yes	41	64.1	19	37.3	60	52.2
No	23	35.9	32	62.7	55	47.8

(*) 13 children died before the age of 2 years and 10 children after the age of 2 died;

(**) 37 dossiers did not furnish this information;

(***) 11 dossiers did not furnish this information,

(****) 9 dossiers did not furnish this information.

It was observed that with regard to the sex of children in the infected group, 34 children (47.9%) were male and 37 (52.1%) were female. In the case of non-infected children, 31 (58.5%) were males and 22 (41.5%) were females. During the survey period, the follow-up service at the Pediatric Immunodeficiency Department was regularly used by 32 infected children (45.1%); 13 children (18.4%) abandoned the follow-up; 3 children (4.2%) were transferred to another service and 23 children (32.4%) died. Thirteen of the children (56.5%) who died did so before the age of 2, and 10 children (43.4%) died after the age of 2. The infected children reported at the clinic according to the evolution of the disease, which was usually weekly. In the group of non-infected children, 30 children (56.6%) had a regular follow up, 10 children (18.8%) abandoned the program, 1 child (1.9%) was transferred to another program and 12 (22.6%) children were discharged. None of the children from this group died. Most of the children came from a background of low income and mother's education.

Table 2 shows the distribution of the children in the infected and non-infected groups with regard to the weight and length at birth and gestational age.

**Table 2:** Infected and non-infected children according to their weight and length at birth and their gestational age

Children	Weight at birth (kg)	Length at birth (cm)	Gestational age (weeks)
Infected	n = 59*	n = 36*	n = 29*
Mean	2.95	48.6	39.4
SD	0.69	2.85	1.70
Median	2.90	48.5	40.0
Non-infected	n = 38*	n = 33*	n = 25*
Mean	2.95	48.6	39.3
SD	0.69	2.62	1.98
Median	2.92	49.0	40.0
Total (n = 124)	n = 97*	n = 69*	n = 54*
Mean	2.95	48.6	39.3
SD	0.68	2.72	1.82
Median	2.90	49.0	40.0

* Patients whose information was available; sd = standard deviation.

It was verified that the weight and length at birth and the gestational age were similar for the two groups.

The results of the Z-score analyses are shown in Table 3. It can be seen that there is a difference between all the variables for both groups except in the case of length/age at 3 months and weight/length at 21 months as the value obtained for P was > 0.05.

**Table 3:** Z-score values for the infected and non-infected groups of children according to the anthropometric index and age

Group Z-score	Children								P-value
	Infected				Non-Infected				
	n	Mean	SD	Median	n	Mean	SD	Median	
H/A - 3 months	21	-1.37	1.16	-1.35	30	-0.98	1.30	-0.78	0.1652 (**)
H/A -months	25	-2.46	1.16	-2.49	28	-0.91	1.26	-0.89	0.0000(*)
H/A - 15 months	28	-2.59	1.46	-2.76	29	-1.06	0.96	-0.88	0.0000(*)
H/A - 21 months	37	-2.12	1.48	-2.51	25	-0.59	1.19	-0.37	0.0001(*)
W/H - 3 months	21	-0.39	0.82	-0.62	30	0.73	1.87	0.31	0.0005 (*) (**)
W/H - 9 months	25	-0.95	1.17	-0.76	28	-0.03	0.99	0.10	0.0033(*)
W/H - 15 months	28	-0.86	1.06	-0.86	29	-0.22	1.04	-0.06	0.0249(*)
W/H - 21 months	37	-0.66	1.25	-0.57	25	-0.39	1.07	-0.62	0.3747
W/A - 3 months	22	-1.35	1.05	-1.37	30	-0.47	1.15	-0.41	0.0067(*)
W/A - 9 months	26	-2.50	1.35	-2.40	28	-0.78	1.07	-0.61	0.0000(*)
W/A - 15 months	28	-2.28	1.38	-2.37	29	-0.85	0.85	-0.93	0.0000(*)
W/A - 21 months	39	-1.69	1.46	-1.75	26	-0.62	1.10	-0.85	0.0030(*)

(*) There is evidence that a difference exists;

(**) The Mann-Whitney test was used; H/A = length/age; W/H = weight/length; W/A = weight/age.

## DISCUSSION

The analysis of the growth of children born to HIV+ mothers reveals the social and affective factors involved in these children's families. Children of HIV+ mothers are raised in an environment highly affected by the disease and the circumstances that follow it. The children that develop infection have a path of highs and lows depending of the availability of the treatment and the response that each patient presents to this treatment. This work refers to children treated before the recently proposed drug therapy, and the objective of this study was not, at this time, to evaluate the effect of clinical treatment on the patients.

HIV can be transmitted vertically during pregnancy, at birth and or during breast feeding. According to the clinical data, at least 50% of the transmissions seem to occur at birth. This supposition is based on the fact that in the case of most children, it is difficult to detect viral particles during the first weeks of life without the viral multiplication or the formation of antibodies which occurs around the second month.^17^ It is probably at this stage that the virus comes out of latency and begins the phase of viral replication which is proportional to the stimulus offered by the immune system.

Children born to mothers who are seropositive for HIV, are usually born seropositive because of the passage of antibodies of class IgG through the placenta. The maternal antibodies may remain detectable until the 15th month of life and as a result, the ELISA and the Western Blot tests do not diagnose HIV infection.

A definite diagnosis of infection in children younger than 15 months old can only be done by directly researching the virus or its components: viral cultures, the presence of antigenemia (antigen P24), the polymerase chain reaction (PCR) and viral load.^1^

The growth of children born to mothers infected with the human immunodeficiency virus was evaluated in this study. Those who developed the infection were compared to the seroreversed children. Both these groups belonged to the same social-economic class. It was observed that the growth of the infected children was already compromised at 3 months. During the first year of life they were proportionally smaller in weight as well as length, although at 3 months the mean length was less in the HIV positive group than in the seroreversed group. At that stage, the difference was still not significant, but showed that weight was compromised earlier than length.

Weight and length measurements have traditionally been used as criteria for determining the nutritional state and health of children.^18^ Children infected by the human immunodeficiency virus usually have nutritional problems and the “wasting” syndrome was described as a condition that indicated AIDS in 17% of the children in 1994.^19^ Oleske, et al.^20^ (1983) and Rubinstein et al^21^ (1983) have highlighted growth retardation and loss in weight in early reports on children with AIDS, although very little progress has been made during recent years in understanding the mechanism of these observations.

It was observed that there was no difference regarding weight and length at birth and the gestational age between infected and non-infected children. Other studies that have analyzed the growth of HIV infected children have also confirmed the above observation,^10,22,23^ but there are other researchers who have found differences between the groups.^24^

Abnormalities in the development of the child most probably begin after birth and take place rapidly, even in asymptomatic children. Growth models are not always foreseeable, as the clinical course of the disease varies.

Other researchers have found that during the prenatal period, the children infected by the virus showed a reduction in weight/age and length/age when compared with non-infected children during the first four months of life. Reductions in weight and length were proportional resulting in a nearly normal relationship – weight/proximal length.^11^ In this study, it was observed that the difference in weight when compared with length diminished at 15 months and was not significant at 21 months. With time, length was compromised and the relationship weight/length modified.

The failure to grow is reported in approximately one third of the children infected by the human immunodeficiency virus and is associated with a reduced rate of survival. Loss of weight occurs in the first months of life before there is a decline in the length of children born to mothers infected with the virus. In long term survivors, the reduction in growth continues and may be associated with reduced body mass.

Moye, et al.^13^ (1996) showed that infected and non-infected children born to infected mothers had similar weight, length and gestational age at birth. However, at the age of 2 months, the infected children revealed a significant reduction in the circumference of the head and in weight, but the length did not diminish significantly until the age of 4 months. These differences were maintained for 18 months.

As the children became more symptomatic, diarrhea, malabsorption and enteric infections became more common and growth was affected.

In short, during the first two years of life, the Z-scores for infected children regarding weight and length in the population studied were less than those for non-infected children. The growth of the children was proportional but their size was inferior when compared to their chronological age. HIV affects the infected children rapidly and is demonstrated by premature growth retardation.

## CONCLUSION

These results have helped us to reach the conclusion that there was a significant difference with regard to weight and length between the group of infected children and that of non-infected children. On analyzing the Z-score it was observed that there was a significant difference between the two groups with regard to the following indices: length/age at 9, 15 and 21 months; weight/length at 3, 9 and 15 months and weight/age at 3, 9, 15 and 21 months.
